# Preparation and Properties of Electrospun Cellulose Acetate Fibers Containing Rosemary, Clove, and Thyme Essential Oils

**DOI:** 10.3390/molecules31142533

**Published:** 2026-07-21

**Authors:** Ramune Rutkaite, Vesta Navikaite-Snipaitiene, Deimante Rosliuk, Ilona Jonuskiene, Jonas Matulevicius, Zaneta Rukuiziene, Asta Tamuleviciene, Valdas Jakstas, Liudas Ivanauskas

**Affiliations:** 1 Department of Polymer Chemistry and Technology, Kaunas University of Technology, Radvilenu Rd. 19, LT-50254 Kaunas, Lithuania; vesta.navikaite@ktu.lt (V.N.-S.); deimante.rosliuk@ktu.lt (D.R.); 2 Department of Organic Chemistry, Kaunas University of Technology, Radvilenu Rd. 19, LT-50254 Kaunas, Lithuania; ilona.jonuskiene@ktu.lt; 3 Department of Environmental Technologies, Kaunas University of Technology, Radvilenu Rd. 19, LT-50254 Kaunas, Lithuania; jonas.matulevicius@gmail.com; 4 The Centre of Laboratories at the Faculty of Mechanical Engineering and Design, Kaunas University of Technology, Studentu Str. 56, LT-51424 Kaunas, Lithuania; zaneta.rukuiziene@ktu.lt; 5 Institute of Materials Science, Kaunas University of Technology, K. Baršausko Str. 59, LT-51423 Kaunas, Lithuania; asta.tamuleviciene@ktu.lt; 6 Department of Pharmacognosy, Lithuanian University of Health Sciences, Sukileliu Ave. 13, LT-50162 Kaunas, Lithuania; valdas.jakstas@lsmu.lt; 7 Institute of Pharmaceutical Technologies, Lithuanian University of Health Sciences, Sukileliu Ave. 13, LT-50162 Kaunas, Lithuania; 8 Department of Analytical and Toxicological Chemistry, Lithuanian University of Health Sciences, Sukileliu Ave. 13, LT-50162 Kaunas, Lithuania; liudas.ivanauskas@lsmu.lt

**Keywords:** electrospinning, cellulose acetate, essential oils, antioxidant activity, active packaging

## Abstract

Single-needle electrospinning and needle-free electrospinning were applied to fabricate cellulose acetate (CA) fibrous mats containing rosemary (RO), clove (CL) and thyme (TH) essential oils (EO) from binary or ternary solvent mixture solutions. When an acetone, dichloromethane, and dimethylformamide mixture was used as the solvent system, cylindrical nanofibers with an average diameter between 255 and 617 nm were obtained. Removing dimethylformamide from the spinning solution resulted in flattened microfibers with significantly larger diameters, varying from 1242 to 2939 nm. Furthermore, CA nanofibers loaded with RO, CL or TH essential oils with diameter distributions ranging from 55 to 176 nm, from 69 to 215 nm and from 44 to 291 nm, respectively, were produced from ternary solvent mixture solutions using needle-free electrospinning equipment. FTIR and TGA studies revealed the incorporation of essential oils into the fiber structure, and GC-MS was used to evaluate the release of bioactive components from fibrous mats. The electrospun nanofibers of CA/RO, CA/CL and CA/TH showed excellent antioxidant activity and antimicrobial properties against *Escherichia coli*, *Pseudomonas aeruginosa* and *Listeria monocytogenes*. In addition, the CA/CL fibrous mat was tested in the storage of fresh beef steaks.

## 1. Introduction

Electrospinning has emerged as a valuable technique for the fabrication of nanofibrous structures that are continuous, lightweight, and possess a high porosity with an extensive surface area [1]. These structural characteristics enable efficient encapsulation, protection, and controlled release of active compounds, making electrospun materials highly attractive for applications in tissue engineering [2], wound healing [3], pharmaceutical delivery systems [4], enzyme stabilization [5], and active food packaging [6]. A key benefit of this technique is the straightforward integration of functional additives into the fiber matrix. In food packaging, electrospun nanofibers can act not only as passive barriers but also as active functional systems capable of incorporating oxygen scavengers [7], antimicrobial compounds [8], thermal indicators [9], ethylene scavengers [10], or even components controlling CO_2_ release [11]. These functionalities allow the packaged foods to better resist spoilage and extend their shelf life.

Recent advances in active food packaging technologies have increasingly focused on replacing petroleum-based packaging materials such as polyethylene (PE), polypropylene (PP), and polyethylene terephthalate (PET) with biodegradable polymers such as polycaprolactone, polyvinyl alcohol, gelatin, zein, and derivatives of various polysaccharides such as chitosan and cellulose acetate (CA) [12]. However, electrospinning polysaccharides can be challenging because their strong hydrogen bonding often results in high solution viscosity or gelation. Therefore, selecting an appropriate solvent system and polymer concentration is critical for successful electrospinning of polysaccharides [13]. Despite these challenges, electrospun CA fibers have attracted increasing attention due to their renewability, biodegradability, and versatile material properties [14,15,16].

Considerable efforts have been devoted to optimizing solvent systems and processing conditions for CA electrospinning. Previous studies demonstrated that solvent composition significantly affects fiber diameter, bead formation, pore generation, and solvent evaporation kinetics. Electrospinning of CA has been investigated using dioxane, dichloromethane (DCM), dimethylformamide (DMF), chloroform, N,N-dimethylacetamide (DMA), ethanol, acetone, and mixed solvent systems [17,18]. Tungprapa et al. investigated that both polymer concentration and solvent choice have a strong impact on the ability to electrospin CA solutions. A mixture of acetone and DMA at a 2:1 volume ratio has been identified as a particularly effective solvent system, enabling stable electrospinning of CA solutions at concentrations of 12.5–20% [19]. Similarly, highly volatile binary DCM/acetone systems generated porous and bead-free fibers due to the combined effects of solution viscosity and rapid solvent evaporation [20]. Furthermore, a ternary solvent system of acetone/DMF/trifluoroethanol was used to prepare protein A/G-functionalized regenerated cellulose nanofibers, which served as affinity membranes for the purification of immunoglobulin G [21]. Such structural variations are particularly important in active packaging because fiber diameter and porosity govern the diffusion of encapsulated compounds, thereby affecting release kinetics, antimicrobial and antioxidant efficiency. Smaller fiber diameter and more porous morphology generally accelerate the migration of bioactive molecules, while denser and thicker fibers tend to provide slower and more sustained release.

The incorporation of bioactive compounds, including essential oils (EOs), vitamins, drugs and others, into electrospun CA fibers has recently attracted considerable interest for the development of functional materials [22,23,24,25,26]. EOs are especially promising due to their strong antimicrobial, antifungal, larvicidal, insecticidal and antioxidant activities, as well as their classification as Generally Recognized as Safe (GRAS) substances by the U.S. Food and Drug Administration [27,28]. Their bioactivity is mainly associated with phenolic constituents capable of disrupting microbial cell membranes, scavenging free radicals, and inhibiting lipid oxidation. Nevertheless, the direct application of EOs in food systems remains limited because of their volatility, hydrophobicity, susceptibility to degradation, and strong aroma, which may adversely affect sensory acceptance when excessive amounts are used. Encapsulation within electrospun fibers offers an effective strategy to overcome these limitations by improving EO stability, reducing rapid volatilization, and enabling controlled release.

Recent studies have demonstrated the considerable antimicrobial and antioxidant potential of EOs in active food preservation systems [29,30,31,32,33,34]. Among them, rosemary, clove, and thyme essential oils are among the most extensively studied food-grade EOs owing to their pronounced antioxidant and antimicrobial properties. Ricardo-Rodrigues et al. summarized that thyme and clove EOs effectively inhibit lipid oxidation and microbial growth in meat products while also contributing characteristic flavor and aroma profiles that are generally well accepted by consumers familiar with these natural seasonings [29]. In addition, Hofmeisterová et al. demonstrated that clove and thyme EOs effectively inhibited bacterial growth and biofilm formation due to the presence of eugenol and thymol in compositions, while Kraśniewska and Gniewosz showed that clove EO incorporated into active packaging coatings improved antimicrobial activity and oxidative stability in food contact applications [30,31]. Furthermore, clove essential oil or eugenol has been incorporated into CA or acrylic/hydrophobically modified starch coatings on corona-treated oriented polypropylene films. The produced films exhibited high antioxidant activity and rapid release of active compounds into the headspace, resulting in their potential as antioxidant packaging materials for fresh meat [32].

Several recent studies have also highlighted that EOs can be successfully incorporated into nanofiber materials through electrospinning. For example, CA nanofibers containing rosemary and oregano essential oils were formed via single-needle electrospinning, demonstrating significant antimicrobial activity against *Staphylococcus aureus*, *Escherichia coli*, and *Candida albicans* [35]. Spasova et al. developed CA electrospun fibers containing rosmarinic acid and reported that the addition of polyethylene glycol promoted a more hydrophilic fiber surface and improved the release of rosmarinic acid, resulting in enhanced antioxidant capacity and significant antifungal activity against *Candida albicans* [36]. However, the fabrication of EO-loaded CA fibers via different electrospinning approaches has not yet been systematically explored.

This study provides a comprehensive comparison of single-needle and needle-free electrospinning technologies for the fabrication of cellulose acetate (CA) fibrous mats incorporating rosemary, clove, and thyme essential oils. These plant-derived essential oils were selected as natural sources of antioxidant and antimicrobial compounds, enabling the development of bioactive fibrous materials. The influence of electrospinning configuration and solvent system composition on fiber generation was systematically evaluated through morphological and structural analyses. The results demonstrated that both the electrospinning technique and solvent composition significantly affect jet formation, fiber diameter, surface morphology, and the occurrence of structural defects. In addition, the presence of essential oils modified the solution characteristics, contributing to variations in fiber formation behavior. The study highlights the importance of optimizing solvent systems to achieve uniform, defect-free fibers while maintaining the functional properties of the encapsulated bioactive agents. The practical application of the fabricated fibrous mats as antioxidant food packaging was validated through their application in fresh beef preservation, demonstrating their potential for biodegradable active food packaging systems. Overall, the findings provide important guidance for the design of scalable, environmentally friendly electrospun materials intended for applications requiring controlled antioxidant and antimicrobial activity.

## 2. Results

### 2.1. Chromatographic Analysis of Essential Oils

The characterization of natural essential oils used in this study was performed by the GC–MS technique. The chromatograms of (clove) CL, rosemary (RO), and thyme (TH) essential oils (Figure S1) revealed that the profiles of dominating volatiles were mostly analogous to those specified in the European Pharmacopoeia (Ph Eur). The analysis of the relative percentage of the areas of separated peaks (area/total area %) in the total ion chromatograms (TIC) demonstrated that the clove essential oil was predominantly composed of eugenol (85.5%), eugenyl acetate (8.3%), and β-caryophyllene (6.1%) and the same as specified in the Ph Eur. The profile of essential oil derived from the flowering parts of RO was characterized by 29.1% of camphor, 22.6% of α-pinene, 20.5% of eucalyptol, 10.5% of camphene, 4.7% of β-pinene, 3.9% of limonene, 2.4% of bornyl acetate, 2.2% of borneol, 1.5% of p-cymene, 0.8% of β-myrcene, and 0.7% of terpineol. Iso-borneol was also identified by mass spectra compared to the libraries; however, a low amount of verbenone was not verified in the tested sample. The tested dominant constituents of RO were the same but lacked complete equivalency in percentage composition compared to Spanish-type rosemary oil specified by Ph Eur. TH contained 53.2% of thymol, 32.1% of p-cymene, 4.5% of linalool, 2.7% of carvacrol, 1.2% of γ-terpinene, 0.9% of β-myrcene, and 0.4% of terpinen-4-ol. The results of the chromatographic characterization have shown that thymol-type thyme essential oil was used in the study. The percentage composition of dominant phytochemicals was very similar to that described in the Ph Eur. In addition, the match of mass spectra to the libraries confirmed the presence of low amounts of alpha-pinene, camphene, eucalyptol and camphor. Meanwhile, alpha-thujene, alpha-terpinene, and carvacrol methyl ether were not confirmed in the tested TH sample.

### 2.2. Fabrication and Characterization of Electrospun Fibrous Mats

Cellulose acetate (CA) and essential oil fibrous mats were successfully electrospun from solutions prepared in ternary or binary solvent mixtures using single-needle or needle-free electrospinning techniques as detailed in the methodology section.

**Figure 1:**
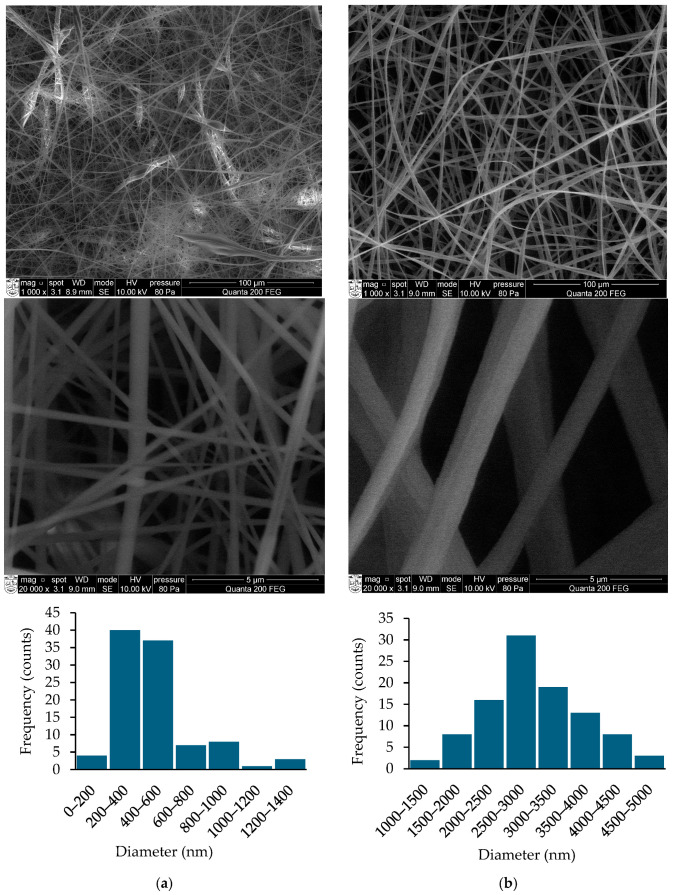
SEM images of electrospun fibrous mats at different magnification (1000×, 20,000×) and fiber diameter distribution diagrams: (**a**) CA1/RO40; (**b**) CA2/RO40.

Firstly, electrospinning of CA and RO fibers was carried out by using an acetone/DCM/DMF ternary solvent mixture with a ratio of 2/1/1 (*v*/*v*/*v*) and by varying RO content in the electrospinning solution using single-needle electrospinning equipment. With the increasing concentration of RO in the electrospinning mixture, a small decrease in both the conductivity and viscosity of the mixture was determined. The SEM images of electrospun mats revealed that CA1/RO fibrous mats were composed of continuous, fine, cylindrical nanofibers with a smooth structure; however, with some defects (beads) (Figure 1a). It is very likely that the formation of the beads was caused by the presence of the less volatile DMF solvent. The average fiber diameter in CA1/RO fibrous mats ranged from around 255 to 623 nm, depending on the fiber composition (Table 1). It could be noted that with the increasing content of RO in the mixture, the average diameter of electrospun fibers was also increasing, as revealed by SEM and statistical analysis. The content of RO in the CA1/RO fibrous mats was found to be in the range from 2 to 15 wt% and was 2–5 times lower compared to feed mixture.

Further, the electrospinning of CA and RO fibers was carried out by using acetone/DCM binary solvent mixture at the ratio 1/1 (*v*/*v*) and again by varying RO content in the electrospinning solution. With the elimination of DMF from the solvent system, the conductivity of the solution decreased by about 5 times, and viscosity was slightly increased. Again, with the increasing concentration of RO in the electrospinning mixture, a small decrease in the conductivity was observed. As can be seen in SEM images of CA2/RO electrospun mats (Figure 1b), elimination of DMF from the electrospinning solution yielded flat ribbon-like and defect-free continuous fibers. Previous studies have revealed that the formation of ribbon-shaped CA fibers is commonly observed when highly volatile solvent systems are used for electrospinning. In these conditions, ribbon-shaped fibers are formed by the collapse of a tube-like fiber skin due to a rapid vaporization of solvents from inside the fiber. As the solvent continues to evaporate, atmospheric pressure causes this tubular structure to collapse, resulting in the characteristic ribbon-shaped morphology [20].

The average fiber diameter in CA2/RO fibrous mats varied from around 1242 to 2939 nm, depending on the fiber composition. With the increasing content of RO in the binary solvent system, the average diameter of the fibers also increased. The content of RO in the CA2/RO fibrous mats ranged from 7 to 13 wt% and was 1.5–3 times lower compared to feed mixture. The absence of fiber defects by the elimination of DMF from the solvent mixture confirms the presumption that slow evaporation of DMF was the main reason for the formation of the beads in the CA1/RO fibrous mats during the electrospinning. However, the obtained CA2/RO fibers were much thicker, as demonstrated by SEM images and fiber diameter distribution diagrams (Figure 1b). Studies have shown that the properties of the electrospinning solution, including viscosity, conductivity, and surface tension, are the main factors that affect how a polymer solution is transformed into electrospun fibers [17]. Although increased viscosity of the electrospinning solutions could have an effect on the formation of thicker fibers, the increase in the average diameter of the CA2/RO fibers was mainly due to decreased conductivity and charge density of the CA and RO solutions in the binary solvent mixture.

**Figure 2:**
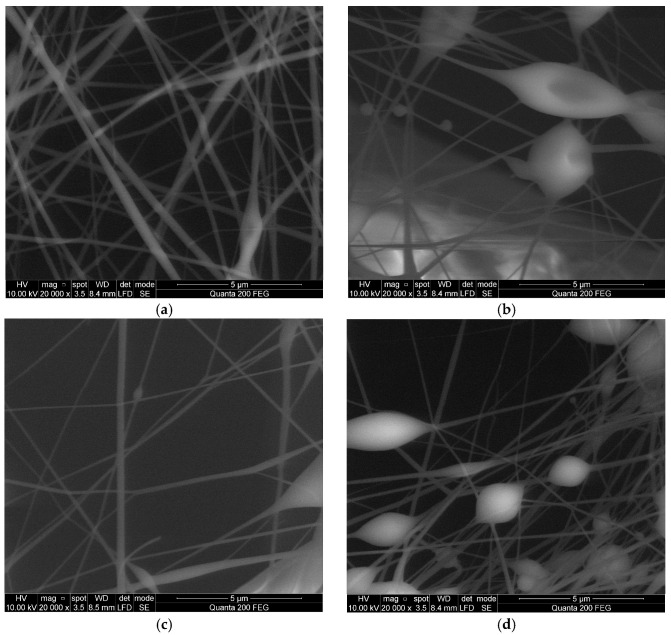
SEM images of the electrospun fibrous mats at magnification of 20,000×: (**a**) CA3; (**b**) CA3/RO40; (**c**) CA3/CL40; (**d**) CA3/TH40.

It was revealed that despite some beads in the electrospun fibers, the formation of thin fibers from a ternary solvent mixture is a more effective way to obtain fibrous mats with a large surface area. Therefore, the fibrous mats of CA and RO, clove (CL) or thyme (TH) essential oils were further produced by using efficient needle-free NanospiderTM electrospinning equipment. The SEM images of electrospun CA3, CA3/RO40, CA3/CL40 and CA3/TH40 samples (Figure 2) revealed that continuous, fine, cylindrical nanofibers were obtained; however, the beads were present in all samples. Although extensive efforts have been made to eliminate bead-on-string structures in electrospun nanofibers through optimization of processing parameters, this morphology may be advantageous for the delivery of bioactive compounds. The beads can serve as reservoirs for bioactives, thereby reducing the initial burst release and promoting sustained release of the active molecules [37]. The fiber diameter in the CA3 fibrous mat ranged from 71 to 323 nm, with a calculated average diameter of 182 nm (Table 1). Meanwhile, the addition of essential oils to the electrospinning solution yielded slightly thinner CA/essential oil fibers with an average diameter varying from 109 to 147 nm. The diameter distribution of CA fibers containing RO, CL and TH essential oils ranged from 55 to 176 nm, from 69 to 215 nm and from 44 to 291 nm, respectively. Finally, it could also be noted that using needle-free electrospinning equipment, the CA and essential oil fibrous mats with much smaller average fiber diameters were produced in comparison with those obtained using the single-needle electrospinning technique. Beyond the reduction of fiber diameter, the primary advantages of needle-free electrospinning over single-needle systems include: (i) the elimination of clogging, as traditional single-needle setups are prone to needle tip blockages caused by rapid solvent evaporation or premature polymer solidification [38]; (ii) substantially higher throughput and seamless scalability, as needle-free systems can generate hundreds to thousands of simultaneous spinning jets from open liquid surfaces or specialized rotating electrodes without the need to balance flow rates across numerous individual capillary channels [39]; and (iii) enhanced spinnability of complex formulations, as open-surface dynamics allow the successful processing of highly viscous fluids, colloidal suspensions, and dispersions that would rapidly clog narrow single-needle orifices [40].

By assessing the hydrophobicity of the obtained fibrous mats (Table 1), it was determined that water contact angles were above 100° for CA1, CA2, and all CA1/RO and CA2/RO samples, pointing toward the hydrophobic nature of the fibrous mats obtained by the single-needle electrospinning setup. It could be noted that by incorporating RO into the fibers, the water contact angle increased by approx. 10–15° and 5–10° for CA1/RO and CA2/RO fibrous mats, respectively, due to the hydrophobic nature of the essential oil. Meanwhile, slightly lower water contact angles were detected for CA3 fibrous mat and fibers containing essential oils obtained by needle-free electrospinning, especially for CA3/RO40 and CA3/TH40 mats, pointing toward the higher porosity of the mats and consequently faster penetration of water drops, despite the hydrophobic nature of CA3/essential oil nanofibers.

**Figure 3:**
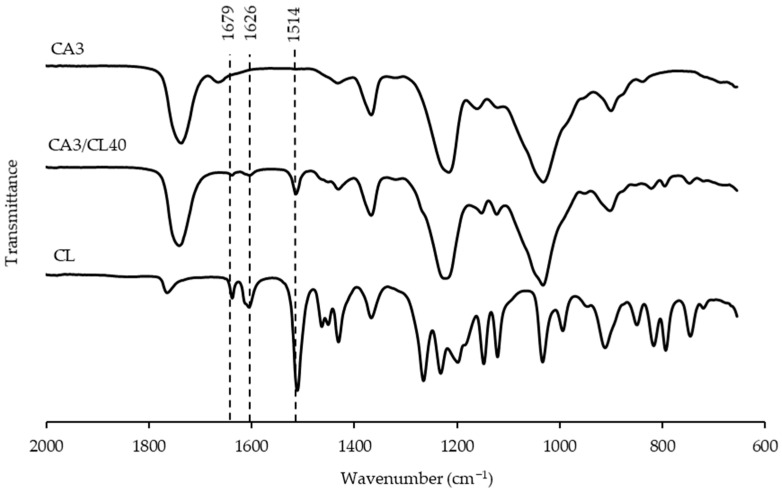
FTIR spectra of clove essential oil, CA3/CL40 and CA samples.

The ATR-FTIR spectroscopy was employed to investigate the composition of the obtained CA/essential oil fibrous mats. Figure 3 represents the comparison of the blank CA3 mat, CL and fibrous mat containing CL (CA3/CL40) spectra. The characteristic bands of CL were at 1679 cm^−1^, 1626 cm^−1^, and 1514 cm^−1^, which indicated the presence of a carbonyl group (C=O) and a vibration peak of a benzene ring, respectively [41]. Other peaks at 912, 817, and 793 cm^−1^ were found from the main CL component eugenol, which are due to the presence of CH2 deformation vibration, stretching vibration of the C-O bond, and bonding vibrations of C-H, respectively. The FTIR spectrum of the CA3 fiber showed characteristic peaks near 1031 and 1218 cm^−1^, attributed to the C-O-C stretching of ether groups. A sharp peak at 1737 cm^−1^ can also be assigned to the C=O stretching of acetyl groups [33]. The CA3/CL40 revealed the peaks relevant to those of both CA3 fiber and CL, indicating the effective incorporation of CL in the fiber structure.

**Figure 4:**
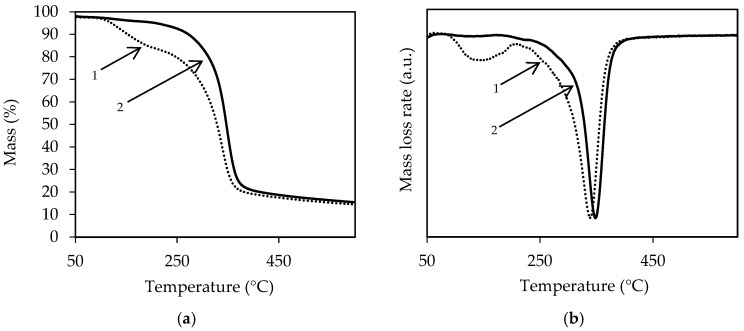
TG (**a**) and DTG (**b**) curves of CA3/RO40 (1) and CA3 (2) fibrous mats.

Thermal analysis was employed to investigate the composition of the obtained CA/essential oil fibrous mats as well as the thermal stability of the electrospun fibers. TG and DTG curves of CA3 and CA3/RO40 samples are depicted in Figure 4. It is obvious that thermal decomposition of CA3/RO40 occurred in two steps; meanwhile, cellulose acetate fiber degradation was characterized by one distinct stage (Figure 4a). The TG curves of both samples also showed a mass loss of about 3% when reaching 100 °C, which could be related to water and solvent residue desorption from the fibrous mats by evaporation. 13% of the mass of CA3/RO40 was lost during the first thermal event due to evaporation of RO volatiles. The second thermal event, which was also characteristic of CA3 fiber degradation, was attributed to the decomposition of cellulose acetate chains. The incorporation of RO into the fibers decreased the thermal stability of electrospun fibrous mats by about 10 °C when compared to CA3 (Figure 4b). The other electrospun fibrous mats presented the same behavior. Therefore, these results confirm that the essential oils were successfully distributed throughout the structure of the electrospun fibers and that their evaporation slightly reduced the thermal stability of the fibers. However, the fabricated fibrous mats are designed to be potentially used as coatings of food packaging films or as packaging components, such as active stickers, rather than as standalone packaging films. Consequently, the observed reduction in thermal stability is unlikely to compromise their functional performance, since it would not affect packaging operations, including heat-sealing processes.

### 2.3. Release Characteristics of Electrospun Fibrous Mats

**Figure 5:**
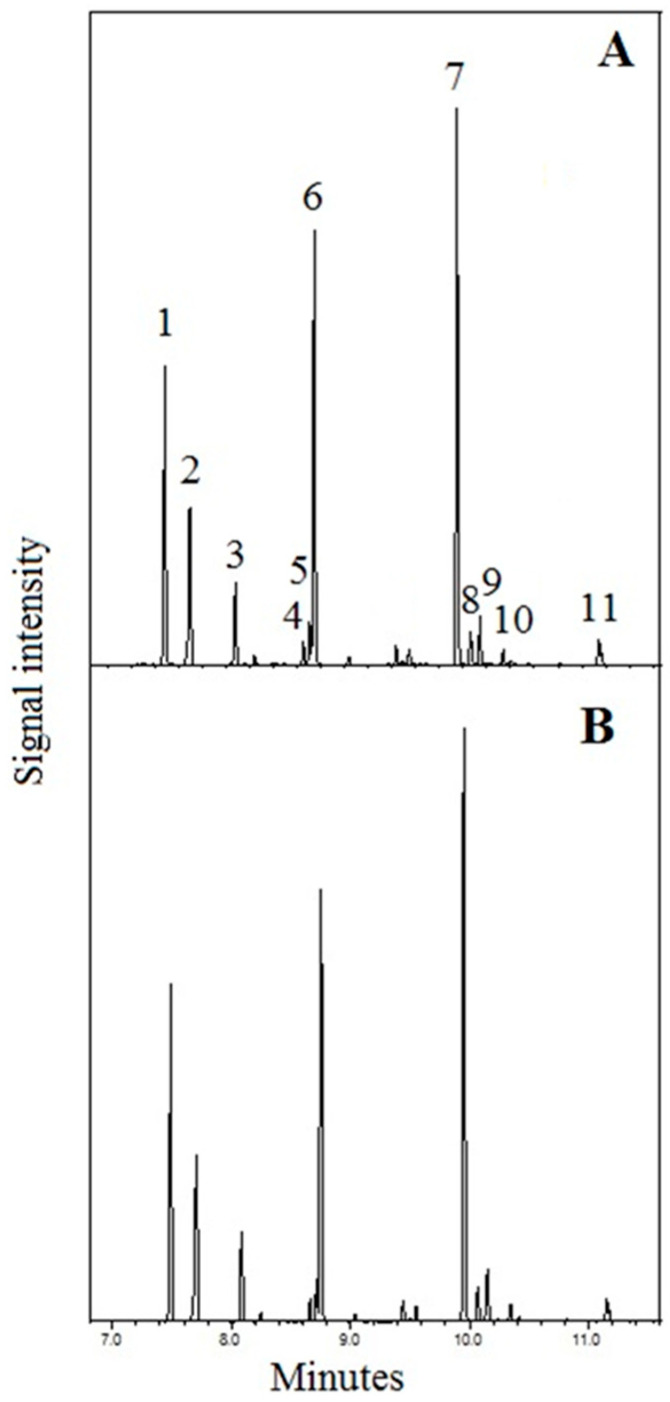
TIC fingerprints of volatile components at initial stage (**A**) and after 14 days (**B**) storage of CA1/RO40 sample: 1—alpha-pinene; 2—camphene; 3—beta-pinene; 4—cymene; 5—limonene; 6—eucalyptol; 7—camphor; 8—isoborneol; 9—borneol; 10—alpha-terpineol; 11—bornyl acetate.

The gas chromatography–mass spectrometry (GC-MS) method was applied for the evaluation of possible alterations of the volatile essential oil ingredient composition of the CA1/RO40 fibrous mat. The total ion chromatographic (TIC) fingerprint profiles and the chromatographic peak areas of the headspace samples were monitored during a 14-day storage period. Fingerprint profiles of volatile oil components released into the environment were identical (Figure 5).

**Figure 6:**
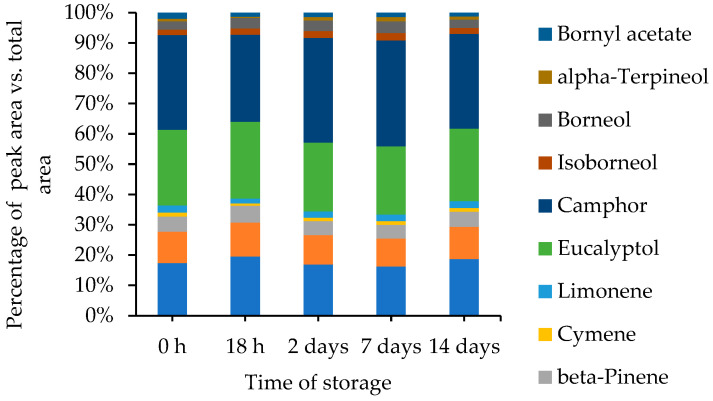
Average distribution of the peak areas of main volatile components in the TIC chromatograms of CA1/RO40 sample during storage period of 14 days.

There were no assignments of additional peaks and peak shape changes during the entire storage period. The variation of percentage distribution profiles of the 11 main volatile oil components was insignificant (Figure 6). Therefore, chemical transformation type instability of bioactive components of tested samples was disallowed, and as a result, possible alterations of the synergistic bioactivity profile during the shelf-life period were unlikely.

**Figure 7:**
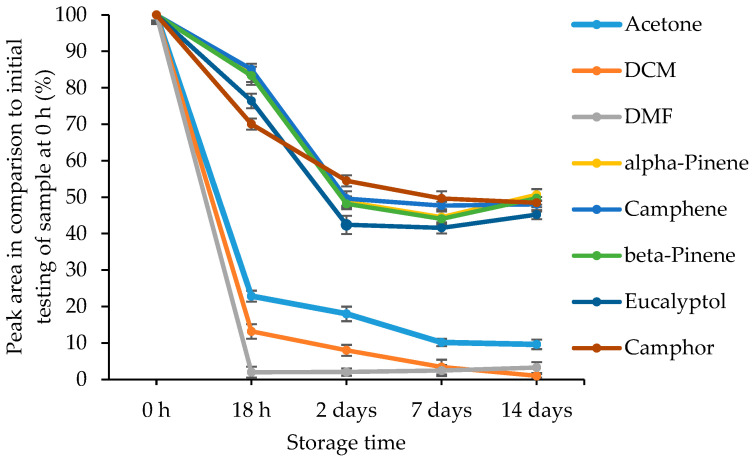
Alterations of the peak area of essential oil volatile components and residual solvents in the selected mass chromatograms of CA1/RO40 sample during storage period of 14 days.

The predominant volatile components of rosemary essential oil (alpha- and beta-pinenes, camphene, eucalyptol and camphor) and residual solvents (acetone, DCM, DMF) were selected for the assessment of the scale of evaporation type instability. The areas of monitored peaks of bioactive markers and residual solvents were processed in the more specific mass ion chromatograms. Peaks of alpha- and beta-pinene and camphene were monitored in the 136 m/z ion chromatogram. Mass ion chromatograms of eucalyptol, camphor, acetone, DCM, and DMF were shaped at 154, 152, 58, 84, and 73, respectively. The peak areas of all monitored volatile components and residual solvents were decreasing during the storage period at different levels (Figure 7).

The release of residual solvents (technological contaminants) into the headspace after 7 days was reduced from approx. 90 percent (acetone) to 98 percent (DCM and DMF). The alteration of releasing of bioactive markers after 7 days has reached the plateau phase, and the residues of bioactive analytes ranged from 42 to 51 percent. The pseudo-second-order kinetic model was used to describe the release kinetics of volatiles during the fiber storage test. The calculated values of the equilibrium release percentage (q_e_) and the pseudo-second-order rate constant (k_2_) are summarized in Table 2 together with the corresponding regression coefficient (R^2^) values.

The obtained R^2^ values ranged from 0.9759 to 0.9999, indicating that the pseudo-second-order kinetic model adequately described the release behavior of both residual solvents and bioactive markers. Residual solvents exhibited the highest equilibrium release percentages, with q_e_ values in the range of 91.61–99.88%, confirming their almost complete evaporation from the fibers during the 14-day period. In contrast, volatiles of essential oils reached considerably lower equilibrium release percentages (53.32–57.55%), indicating that approximately half of the initial amount still remained in the CA1/RO40 fibrous mat after 14 days. DMF exhibited the highest pseudo-second-order release rate constant (k_2_ = 0.3444%^−1^ day^−1^), indicating that equilibrium release was reached rapidly during the initial stage of storage. Acetone and DCM also showed relatively high k_2_ values (0.0621 and 0.0618%^−1^ day^−1^, respectively), whereas the bioactive volatiles exhibited lower k_2_ values (0.0156–0.0413%^−1^ day^−1^), confirming the rapid evaporation of residual solvents while a significant proportion of the bioactive compounds remained incorporated within the CA1/RO40 fibrous mat. Since the bioactive components were stabilized at higher levels than the residual technological contaminants, the developed technology has the potential for controlled release in food environments after a specified maintenance period. The main idea behind releasing bioactive substances in active packaging is to slow down food deterioration and prolong the shelf life. Therefore, adjusting the amount of bioactive compounds over time is beneficial, e.g., a higher release at the beginning of the storage and distribution can help to delay microbial and oxidative damage and preserve the quality of food products, while a lower concentration is preferable by the time the product reaches the consumer. However, a more comprehensive investigation would be required to evaluate the absolute concentrations of remaining residual solvents and the related safety concerns.

### 2.4. Bioactivity of Electrospun Fibrous Mats

The antimicrobial activity of cellulose acetate and essential oil fibrous mats fabricated using needle-free electrospinning was examined against *E. coli*, *P. aeruginosa* and *L. monocytogenes* as test microorganisms. As shown in Table 3, the MIC of the CA3/RO40 fibrous mat against *E. coli*, *P. aeruginosa*, and *L. monocytogenes* was 5 mg/mL, corresponding to 112.5 µg/mL of RO essential oil, which was as effective as the same concentration of free RO essential oil. The MBC of CA3/RO40 fibrous mat against *E. coli*, *P. aeruginosa* and *L. monocytogenes* was 15 mg/mL, which was as effective as approximately 250–375 µg/mL of RO essential oil. Meanwhile, the MIC of CA3/CL40 fibrous mat against *E. coli*, *P. aeruginosa* and *L. monocytogenes* was approximately 5–10 mg/mL, which was as effective as 188–200 µg/mL of CL essential oil. Moreover, it was found that the MBC of CA3/CL40 fibrous mat against *E. coli*, *P. aeruginosa* and *L. monocytogenes* was approximately 10–15 mg/mL, which was as effective as 300–550 µg/mL of CL essential oil. At the same time, the MIC and MBC values of CA3/TH40 fibrous mat against *E. coli*, *P. aeruginosa* and *L. monocytogenes* were 5 and 10 mg/mL, respectively. Although all essential oil-containing mats in this series exhibited some degree of bead formation, particularly CA3/TH40 (see Figure 2a), their antimicrobial performance was not adversely affected, as the observed bioactivity was primarily determined by the chemical structure, physicochemical properties, and release kinetics of the essential oils rather than by morphological imperfections of the mats. Moreover, the beads may serve as reservoirs for the essential oils, enabling the accumulation of greater amounts of bioactive compounds [37]. It may be concluded that the efficient needle-free electrospinning method allows good incorporation of bioactive compounds into the nano- to submicron-structured polymer fibers, which are favorable for the sustained release of antimicrobial agents, i.e., components of essential oils.

The studies of *E.coli*, *P. aeruginosa* and *L. monocytogenes* viability loss in contact with CA3/RO40, CA3/CL40, and CA3/TH40 fibrous mats (Table 4) demonstrated that during a short time of CA3/TH40 nanofiber–bacteria contact just after fiber fabrication, a high level of inactivation for *E. coli* and *L. monocytogenes* was achieved. The CA3/TH40 fibrous mat demonstrated almost complete inactivation of *E. coli* and *L. monocytogenes*, and lower inactivation capacity of around 23% for *P. aeruginosa* was determined. Meanwhile, much lower-level inactivation of *E. coli* and *L. monocytogenes* was achieved in contact with CA3CL40 and CA3RO40 fibers, and the inactivation of *P. aeruginosa* was also slightly lower in comparison to that achieved with CA3/TH40. It can be noted that the CA3/TH40 fibrous mat was the most effective antibacterial material against the tested microorganisms and, therefore, its testing was extended to 14 days. The study demonstrated a high level of inactivation of *E. coli* even after 14 days of storage; meanwhile, complete inactivation of *L. monocytogenes* was still characteristic after 7 days. However, after 14 days, the loss of viability of those bacteria was only approximately 32%. The inactivation of *P. aeruginosa* did not differ much during the testing period of 14 days. However, it is important to note that the latter testing does not fully replicate real-world food packaging conditions, as the active fiber would act through the vapor phase rather than through direct contact. Therefore, future studies should evaluate antimicrobial performance under more practical conditions using the vapor diffusion assay [42] and further explore the interactions with suitable food matrices.

**Figure 8:**
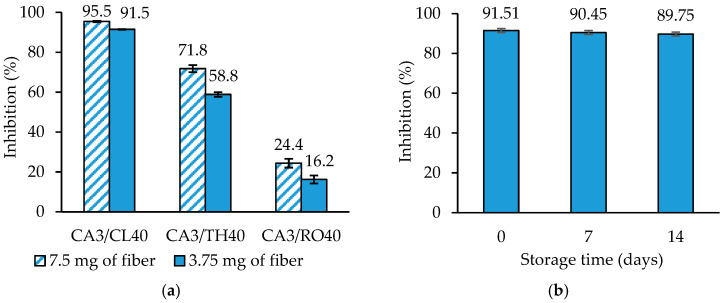
Inhibition (%) of DPPH free radicals: (**a**)—using 7.5 or 3.75 mg of CA3/RO40, CA3/CL40 and CA3/TH40 fibrous mats; (**b**)—changes in antioxidant activity of CA3/CL40 fibrous mat during period of 14 days.

Antioxidant properties of materials, particularly their radical scavenging ability, are very important in food preservation due to the neutralization of free radicals in foods and food packaging environments. Furthermore, free radical scavenging is one of the most important mechanisms by which antioxidants inhibit lipid oxidation [32]. Therefore, the DPPH method is well suited to evaluate the antioxidant activity of natural essential oils and was used to assess the antioxidant effects of electrospun CA fibers containing CL, RO and TH in vitro (Figure 8). It is obvious that the CA fibers containing CL oil showed the highest free radical scavenger activity during the test (Figure 8a). The inhibition of DPPH was 95.5 ± 0.3% and 91.5 ± 0.1%, when 7.5 mg and 3.75 mg of CA3/CL40 fiber were used, respectively. Meanwhile, the antioxidant activity of CA3/RO40 fiber was the lowest. In this case, the inhibition of DPPH ranged from 24.4 ± 2.2% to 16.2 ± 2.0%. Furthermore, CA3/TH40 fiber demonstrated good antioxidant activity with the inhibition of DPPH of 71.8 ± 1.8% and 58.8 ± 1.2%, depending on the concentration of CA3/TH40 fiber used. The antioxidant activity trend observed for the mats containing different essential oils was in good agreement with the results reported by Wang et al. [43], who compared the antioxidant activities of various essential oils and found ABTS^+•^ radical scavenging activities of 97%, 96%, and 27% for clove, thyme, and rosemary essential oils, respectively. This can be attributed to the high eugenol content in clove oil and the high thymol content in thyme oil, both of which are potent phenolic compounds with strong radical scavenging activity. In contrast, rosemary oil predominantly contains camphor, eucalyptol, and α-pinene, i.e., compounds that exhibit lower antioxidant activity. These compositional differences are consistent with the essential oil analysis results presented in Section 2.1. The CA3/CL40 fibrous mat was chosen as the most effective antioxidant material, and to assess the stability of antioxidant properties, antioxidant activity assays of the fiber mat were carried out after 7 and 14 days (Figure 8b). As can be seen from the diagram, the CA fiber containing clove oil presented high stability during all the storage periods. After 14 days, the antioxidant activity of CA/CL fiber decreased only by approx. 1.7%.

### 2.5. Application of Electrospun Nanofibrous Film in Food Packaging

**Figure 9:**
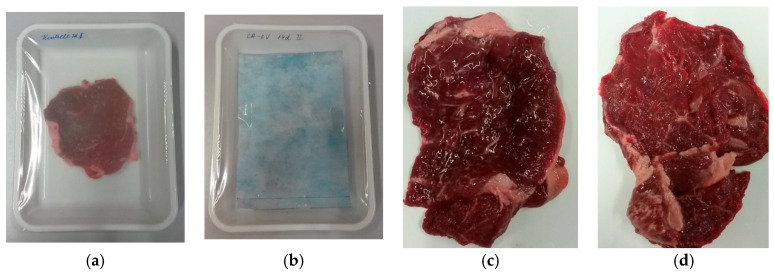
The images of fresh beef steaks packed in the control packaging (**a**) and packaging with CA3/CL40 fibrous mat (**b**), and samples of beef after 14 days of storage in control (**c**) and active (**d**) packaging.

After exhibiting the antioxidant potential of fibers containing essential oils, the fibrous mat containing CL was determined to be the most suitable material for the antioxidant packaging tests. Therefore, polypropylene nonwoven fabric coated with CA3/CL40 electrospun fibrous mat was incorporated in the packaging of fresh beef steaks. Fresh beef is generally stored and commercialized under chilling conditions. Therefore, the changes in the color of packed fresh beef were investigated at 4 °C. As shown in Figure 9, the fresh beef samples were packed in the control (Figure 9a) and CA3/CL40 electrospun fibrous mat containing packaging (Figure 9b) and stored during the period of 14 days.

The changes in the color of fresh beef samples were evaluated during the storage period at each selected time (0, 7, and 14 days) and are presented in Table 5. L*, a* and b* values of beef were 33.10 ± 1.29, 18.52 ± 0.67 and 14.37 ± 0.90 at the start of the experiment, respectively. The L* value indicates a spectrum ranging from black (0) to white (100). No significant changes were observed in L* values of both samples throughout the 14-day storage period, which were around 30 on day 14th. Meanwhile, significant changes in the value of a* (+a* represents red, −a* represents green) were determined for all samples. The control beef sample showed a steady decrease (*p* < 0.05), reaching an a* value of 6.40 ± 0.95 on day 14, indicating deterioration of red color (Figure 9). Meanwhile, beef samples stored in active packaging containing CL maintained better red color stability throughout the storage period, with a* values remaining around 10. These observations correspond with the results of our previous study, in which fresh beef steaks were stored in the packaging with various coatings containing eugenol [32]. As observed, the values of b* (+b* represents yellow, -b* represents blue) of beef samples slightly decreased during storage, remaining at around 10 in both control and active packaging.

Color characteristics of the samples may also be evaluated from hue angle (h_ab_) and chroma (C*) values. Hue values represent the color changes from red to purple. Meanwhile, the chroma value expresses how intense or vivid a color appears [32]. As could be seen, the values of h_ab_ and C* were 37.78 ± 1.29 and 23.44 ± 0.99 on day 0, respectively. After being stored for 14 days, the final values of h_ab_ were 44.90 ± 2.15 and 56.25 ± 3.78 when beef samples were kept in active and control packaging, respectively. Larger hue angles corresponded to a decrease in red coloration. In addition, the vividness of the red color after 14 days was higher (*p* < 0.05) for beef samples kept in active packaging (C* = 14.18 ± 0.98) than in control packaging (C*= 11.55 ± 1.33).

The visual changes in fresh beef steaks were also noticeable at the end of the storage period (Figure 9c,d). As can be seen from the photographs, beef samples stored in active packaging (Figure 9d) retained a bright red color, while beef samples stored in control packaging were much darker, and deterioration of the steak surface was also visible (Figure 9c). It was revealed that meat color changes occur due to the oxidation of red oxymyoglobin into brownish metmyoglobin, with lipid oxidation being the main cause of metmyoglobin formation [44]. Lipid and protein oxidation are often interconnected, with the oxidation of one promoting the formation of compounds that accelerate the oxidation of the other, ultimately contributing to fresh meat discoloration. Meanwhile, the incorporation of antioxidant compounds such as clove essential oil into beef packaging protects against lipid oxidation and may also prevent the oxidation of oxymyoglobin. The release of eugenol and other antioxidant molecules from the electrospun fibrous mat and scavenging of free radicals in the package headspace and in beef samples is suggested as a possible mechanism for preserving the red color of fresh beef steaks in our study. However, other quality attributes of fresh beef, such as lipid oxidation, microbial growth, pH, texture, and sensory characteristics, should be investigated in future studies to fully validate the quantitative and qualitative performance of the active fibrous mat as a component of active packaging.

## 3. Materials and Methods

### 3.1. Materials

Cellulose acetate (CA) (average Mn~30,000 by GPC, acetyl content 39.8 wt. %), rosemary (RO), thyme (TH) and clove (CL) essential oils, 2-2-diphenyl-1-picrylhydrazyl (DPPH), dichloromethane (DCM) (anhydrous, ≥99.8%), N,N-dimethylformamide (DMF) (anhydrous, 99.8%), and methanol (anhydrous, 99.8%) were purchased from Sigma-Aldrich Chemie GmbH (Schnelldorf, Germany) and used without further purification. Acetone (≥99.8%) was purchased from Reachem Slovakia s.r.o (Bratislava-Petrzalka, Slovakia). *Escherichia coli* (ATCC 25922), *Pseudomonas aeruginosa* (ATCC 19429), and *Listeria monocytogenes* (ATCC 19111) were obtained from Leibniz Institute DSMZ-German Collection of Microorganisms and Cell Cultures (DSMZ) (Braunschweig, Germany).

### 3.2. Chromatographic Analysis

Evaluation of the volatile components of essential oils was performed by GC–MS technique on an RXI-5MS (Restek Corporation, Bellefonte, PA, USA) column (30 m × 0.25 mm I.D. and film thickness of 0.25 μm) using helium (99.999% purity) as the carrier gas. A GC2010 Plus gas chromatograph (Shimadzu technologies, Kyoto, Japan) was interfaced with a GCMS-QP2010 (Shimadzu, Japan) mass selective detector. Clove essential oil (CL) was analyzed using a temperature program that started at 80 °C (initially held for 1 min), followed by a temperature increase of 8 °C min^−1^ to 170 °C and then of 30 °C min^−1^ to 270 °C. Injections were made in split mode; the split ratio was 50. A constant column flow rate of carrier gas was 1.44 mL/min, the injection temperature was set at 100 °C, the ion source temperature was held at 200 °C, and the interface temperature was held at 250 °C. The analysis of rosemary essential oil (RO) volatiles started at 70 °C (hold for 10 min), followed by a temperature increase of 20 °C min^−1^ to 250 °C, the split ratio was 30, the column flow rate was 1.19 mL/min, and injection was set at 100 °C, ion source at 200 °C, and interface at 280 °C. The thyme essential oil (TH) analysis program was set to 50 °C (hold for 2 min), followed by a temperature increase of 2 °C min^−1^ to 215 °C; the split ratio was 50, column flow—1.22 mL/min, and the injection ion source and interface temperature were the same as for RO. Mass spectra of ionized fragments in the range from 35 to 500 *m*/*z* were collected after electron impact ionization (70 eV). Data were acquired and processed using the LabSolutions Insight software 5.2 (Shimadzu Technologies, Kyoto, Japan). The identification of separated components was performed by comparison with the chromatographic retention characteristics of the mass spectral libraries FFNSC2 and NIST14, and based on European Pharmacopoeia (Ph. Eur.) monographs: Clove oil (01/2008:1091 corr. 7.6), Rosemary oil (01/2008:1846), and Thyme oil, thymol type (01/2012:1374 corr. 10.0).

### 3.3. Preparation and Characterization of Electrospinning Solutions

Solutions of CA and essential oils (EO) were prepared in a ternary mixture of acetone: DCM: DMF (2:1:1 by volume) or a binary mixture of acetone: DCM (1:1 by volume) under constant stirring at room temperature following the procedure described in previous papers [20,45]. The homogeneous solutions were prepared by first dissolving CA and introducing EO (RO, CL or TH) into the solvent mixture. The CA concentration in all solutions was 11 g/L, and different concentrations of EO were employed to prepare different samples. The detailed compositions and characteristics of prepared solutions are listed in Table 6.

The samples prepared in ternary solvent mixture and binary solvent mixture and submitted to single-needle electrospinning are denoted as CA1/EO and CA2/EO, respectively. Meanwhile, the samples prepared in a ternary solvent mixture and containing 11 g/L of CA and 7 g/L of essential oil (RO, CL or TH) were submitted to needle-free electrospinning and are indicated as CA3/EO.

Before electrospinning, shear viscosity was measured for all the solutions using a rotational viscometer RheoTec RC 01/02 (Bad Driburg, Germany) equipped with a small-sample thermostated adapter and spindle TR8 at 25 ± 0.1 °C, while electrical conductivity measurements of the spinning solutions were performed with a conductivity meter Radelkis OK-102 (Budapest, Hungary).

### 3.4. Electrospinning

#### 3.4.1. Fabrication of Cellulose Acetate and Rosemary Essential Oil Fibrous Mats by Single-Needle Electrospinning

**Figure 10:**
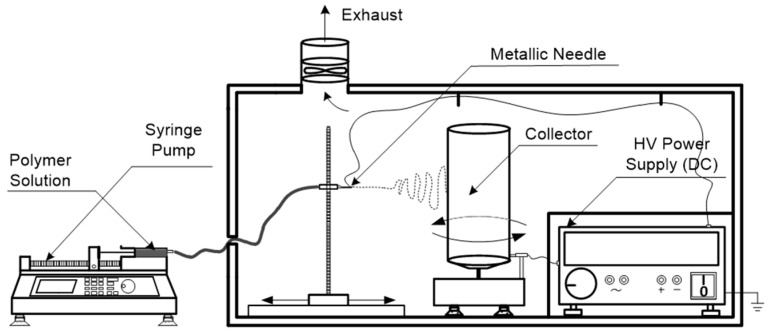
Scheme of the single-needle electrospinning setup used for fabrication of CA1/RO and CA2/RO fibrous mats.

The fabrication of CA and RO fibrous mats (CA1/RO—obtained from a ternary solvent mixture, and CA2/RO—obtained from a binary solvent mixture) has been carried out in a controlled single-needle electrospinning setup, as shown in Figure 10.

The electrospinning device included a high-voltage DC power supply based on the “Flyback” principle, refs. [46,47], and a fiber collector of electricity grounded aluminum foil that covered a vertically positioned roller at a linear speed of 0.025 m/s (rotation frequency of 6 rpm). Tip-to-collector distance was 17 cm, and a positive high voltage of 18 kV was applied to the needle of a syringe pump (LSP01-1A, Baoding Longer Precision Pump Co., Ltd., Baoding, China) for all experiments. The solution feed rate of 3 mL/h was held constant, and the electrospinning duration was 1 h for all experiments. The humidity level and ambient temperature of the electrospinning chamber were maintained at 40% and 23 °C, respectively.

#### 3.4.2. Fabrication of Cellulose Acetate and Rosemary Essential, Clove and Thyme Essential Oil Fibrous Mats by Needle-Free Electrospinning

The fabrication of CA and RO, CL, and TH essential oil fibrous mats, namely, CA3/RO, CA3/CL and CA3/TH, have been achieved by using NanospiderTM NS Lab 200 equipment (Elmarco s.r.o., Liberec, Czech Republic) with a rotational cylindrical electrode with tines, by means of the electrospinning process, proceeded by stretching the polymeric solution from the rotational electrode (immersed in bath with a spinning solution) to the upper electrode, as described previously [48]. The fiber web was formed on polypropylene (PP) spunbonded nonwoven fabric (with an aerial mass of 21.5 g/m^2^, and 20 µm diameter of the fibers). The speed of supporting material was 0 m/min. The distance between the electrodes was 17 cm, and the voltage applied was 70 kV. The temperature of the electrospinning environment was 23 ± 2 °C, and the air humidity was 40 ± 2%.

### 3.5. Characterization of Electrospun Fibrous Mats

FT-IR spectra of the fibrous mats were recorded by using a Frontier FT-IR spectrometer (Perkin Elmer, Waltham, MA, USA) with a single reflectance horizontal ATR (Attenuated Total Reflectance) cell equipped with a diamond crystal. The data were recorded in the spectral range of 655 to 2000 cm^−1^ by accumulating 5 scans with a resolution of 4 cm^−1^.

The thermogravimetric analysis was performed with a Perkin Elmer TGA 4000 instrument. The TG measurements were carried out at a heating rate of 10 °C·min^−1^ from 35 to 600 °C under a nitrogen atmosphere (flow rate 20 cm^3^·min^−1^). About 10 mg of sample was loaded into the ceramic pan. The mass loss at 200 °C was used to determine the amount of essential oils in the electrospun fibers. The mass variation event corresponding to the degradation of the essential oil was analyzed, and the load was calculated using the method described by Barbosa et al. [49].

A drop shape analyzer DSA 25 (Krüss GmbH, Hamburg, Germany) was used for determining the contact angle of the samples. The instrument was composed of a halogen light source, a camera with a 656 × 492 pixel resolution and a zoom/focus (up to 6.5 times) mode, and a manual table movable in the x-, y- and z-axes, onto which solid samples were placed. The sessile drop method was used to measure the contact angle. The creation of drops of the distilled water was assured using an automatic single-dosing unit, and 2 μL volume drops were deposited on the analyzed surface. The contact angle was measured at 3 different points on each sample. The contact angles were determined with the original software Advance version 1.1.0.2 (Krüss GmbH, Hamburg, Germany) devoted for control of the instrument and analysis of the results.

Volatile fractions from fiber samples were collected from the headspace using a SHIMADZU AOC-5000 Plus autosampler and analyzed by the GC–MS technique. The GC–MS system consisted of a SHIMADZU GC2010 Plus gas chromatograph interfaced with a GCMS-QP2010 mass selective detector. The capillary column used was RXI-5MS, with dimensions of 30 m × 0.25 mm I.D. and film thickness of 0.25 μm (Restek Corporation, Bellefonte, PA, USA). Helium (99.999%) was used as the carrier gas at a constant flow of 1.82 mL/min; injection temperature was 100 °C; ion source temperature was 200 °C; interface temperature was 250 °C. The oven temperature was programmed from 35 °C and held for 2 min; a temperature increase of 5 °C min^−1^ was carried out to 50 °C, followed by a temperature increase of 20 °C min^−1^ to 280 °C, followed by an isothermal hold for 2 min. Headspace sampling was performed after sample incubation at 80 °C. Then, 0.300 mL of headspace gas was introduced into the injector using a heated syringe (85 °C). Injections were made in split mode; the split ratio was 60. Mass spectra in the range from 29 to 500 m/z were detected after electron impact ionization (70 eV). Data were acquired and processed with the Lab Solution software (Shimadzu, Japan). Compound identification was performed by comparison with the chromatographic retention characteristics of reference solutions of alkanes and the mass spectral libraries of the GC–MS software (FFNSC2 and NIST14). The experiments were run in triplicate, and the results are presented as average values.

To describe the release kinetics of volatile components and residual solvents from fiber samples during storage, the pseudo-second-order kinetic model was applied and evaluated by calculating the linear regression coefficient (R^2^). The linear equation of the pseudo-second-order model can be represented in the following form:

(1) $$\frac{t}{q_{t}}=\frac{1}{q_{e}}\cdot \;t\;+\;\frac{1}{k_{2}\cdot q_{e}^{2}}$$

where q_t_ (%) is the percentage of volatile component or residual solvent released at time t (day), q_e_ (%) is the equilibrium percentage of volatile component or residual solvent released, k_2_ (%^−1^·day^−1^) is the pseudo-second-order rate constant. The equilibrium release percentage q_e_ was established from the slope of the plot t/q_t_ versus t, and the rate constant k_2_ was determined from the intercept.

The structure of the electrospun fibrous mats was analyzed by using a scanning electron microscope (SEM) Quanta 200 FEG (FEI, The Netherlands). Fiber diameter distribution was determined with Image J1.50 software (National Institute of Health, Rockville Pike, MD, USA) by measuring 100 random fibers from micrographs representative of fiber morphology. Average fiber diameter and standard deviation were calculated.

### 3.6. Antimicrobial Studies

#### 3.6.1. Screening of Antibacterial Activity of Essential Oils

The minimal inhibitory concentration (MIC) was defined as the lowest concentration of essential oils (RO, CL or TH) inhibiting the visible growth of bacteria *E. coli*, *P. aeruginosa* and *L. monocytogenes*. The essential oil was incorporated into broth medium (peptone, meat extract for *E. coli* and *P. aeruginosa*; yeast extract, tryptic soy broth for *L. monocytogenes*) tubes to obtain the final concentrations varying from 6.25 to 500 µg/mL. One-hundred microliters of standardized suspension of each tested organism (107 CFU/mL) was transferred to each tube. The tubes were incubated at 37 °C in a shaking incubator for 24 h. The lowest concentration of the tested sample at which the visual growth of the tested microorganisms was inhibited was determined as MIC. Furthermore, the concentrations showing complete inhibition of bacterial growth were identified, and 100 μL of each culture broth was transferred onto the agar plate and incubated for a specified time and temperature, as mentioned above. The complete absence of growth of microorganisms on the agar surface by using the lowest concentration of the sample was defined as the minimum bactericidal concentration (MBC).

#### 3.6.2. Evaluation of Antibacterial Activity of Electrospun Fibrous Mats

The dilution method was applied to quantitatively determine MIC and MBC of fibrous mats containing RO, CL and TH [50]. Firstly, the tested microorganism was inoculated into 50 mL of nutrient broth in order to obtain a concentration of approximately 1.5 × 10^8^ CFU/mL and then adjusted to approximately 10^6^ CFU/mL with nutrient broth. Subsequently, 250 mg of the respective fibrous mat was added to 3 mL of co-solvent (mixture of ethanol:water at 1:1 *v*/*v*), and essential oil was extracted into the solution by ultrasonic shaking for 1 h. Then, the sample was further diluted with co-solvent (mixture of ethanol:water at 1:1 *v*/*v*) to prepare a series of solutions with varied concentrations (0.5; 1; 2.5; 5; 10; 15; 20 mg/mL). After that, 0.5 mL of each dilution was added into a tube containing 0.1 mL of the above inoculum and 4.4 mL of nutrient broth. Negative control was performed by the addition of 0.5 mL of co-solvent (mixture of ethanol:water at 1:1 *v*/*v*) into 0.1 mL of inoculum and 4.4 mL of nutrient broth, and blank control was carried out by adding 0.1 mL of inoculum into 4.9 mL of nutrient broth. All the tubes were incubated at 37 °C for 24 h. After that, 100 µL aliquots of solution were withdrawn from the tubes without visible turbidity and plated onto agar by the pour-plate method. The colonies in the plates were counted after incubation at 37 °C for 24 h. All tests were performed in triplicate.

The viability loss of *E. coli*, *P. aeruginosa*, *L. monocytogenes* in contact with CA3/RO40, CA3/CL40 and CA3/TH40 was assayed according to a modified ASTM shake flask method [51]. Electrospun mat (16 mm × 125 mm) was placed into a culture tube along with 4 mL of *E. coli*, *P. aeruginosa* or *L. monocytogenes* suspension (5 × 10^6^ cells/mL) in an isotonic solution (0.9% NaCl, pH 5.7). The samples were shaken at 200 rpm in an orbital shaker maintained at 37 °C. After 30 min, 100 µL was pipetted out from the tubes, and serial dilutions were plated onto LB plates using the spread plate method. After 24 h incubation on agar plates, the number of viable bacteria was counted then multiplied by the dilution factor and expressed as the mean colony forming unit (CFU) per mL. The percent reduction of bacteria resulting from contact with the electrospun fiber mats was determined using the following equation:

(2) $$Bacteria\;reduction\;(\%)=\frac{B-A}{B}\times 100$$

where A represents the mean log10 density of bacteria for the sample containing the substrate coated with electrospun fibers and B represents data for the untreated substrate.

### 3.7. In Vitro Antioxidant Activity Assessment

The antioxidant properties of electrospun fibers were assessed by measuring the disappearance of the purple color of the methanol solution of DPPH. 7.5 mg or 3.75 mg of CA3/EO fibrous mat was added to 5 mL of 0.1 mM DPPH solution and kept under stirring (300 rpm) in the dark for 30 min. The absorbance of DPPH solution at 517 nm was measured using a UV/Vis spectrometer T60 (PG Instruments, Wibtoft, Great Britain). The CA3 fibrous mat (mat without EO) in DPPH solution was used as a negative control, and methanol was used as a blank. The inhibition of DPPH radicals was calculated by using the following formula:

(3) $$I(\%)=\frac{A_{0}-A_{s}}{A_{0}}\times 100$$

where A_0_ is the absorbance of the initial DPPH solution at 517 nm, and A_S_ is the absorbance of the DPPH solution exposed to the fibrous sample.

The stability of the antioxidant activity of the CA3/CL40 fibrous mat was investigated using the same DPPH method described above. The sample was stored in sealed bags at 18 ± 2 °C for 14 days.

### 3.8. Preparation and Assessment of Antioxidant Packaging for Fresh Beef

The fresh beef steaks (samples from longissimus thoracis muscle) of about 70 g in weight (1.5 cm thick) were cut using a knife. Each steak was placed into a PET tray of 21.4 × 16.0 × 3.5 cm. The fresh beef samples were packed under normal (air) atmosphere and sealed with PET/PE/EVOH/PE 57 µm peel film using a tray sealer (Unitronics^®^ M-90). Before the tray sealing, polypropylene nonwoven fabric coated with CA3/CL40 electrospun fibrous mat (204 cm^2^) was attached to PET/PE/EVOH/PE peel film. The packages were kept in a refrigerated cabinet at 4 ± 1 °C in the darkness. Beef samples kept in control packaging (without fibrous mat) and CA3/CL40 fibrous mat-containing packaging were taken at each selected time (0, 7 and 14 days) for color analyses. The color of beef samples was measured at the surface of beef steaks using a colorimeter (BYK Spectro-guide 45/0 gloss, illuminant D65) 30 min after pack opening in order to allow color stabilization on air exposure. The color was characterized by CIE system parameters L*, a* and b*, indicating lightness, redness and yellowness, respectively. The average value for each parameter was the mean of 10 determinations for each. Hue angle (h_ab_) and chroma (C*) parameters were calculated using the following equations:

h_ab_ = tan^−1^(b*/a*) (4)

C* = (a*^2^ + b*^2^)^1/2^ (5)

### 3.9. Statistical Analysis

Data were statistically analyzed by the one-way analysis of variance (ANOVA for Excel, version 2.2). Each experiment was performed in triplicate, and the results were expressed as mean ± SD. Significant differences among the parameter values were determined using Duncan’s multiple range test at a probability level *p* < 0.05.

## 4. Conclusions

Electrospun cellulose acetate/rosemary essential oil fibrous mats were successfully produced using single-needle electrospinning equipment. Using acetone, dichloromethane, and dimethylformamide as solvents in the initial composition, cylindrical nanofibers with an average diameter ranging from 255 to 617 nm were obtained. Meanwhile, the removal of dimethylformamide from the electrospinning solution formed flat microfibers with an average diameter varying from 1242 to 2939 nm. Alternatively, the electrospun nanofibers of CA and rosemary (RO), clove (CL) or thyme (TH) essential oils were produced from ternary mixture solutions by using needle-free NanospiderTM electrospinning equipment, with the diameter distribution ranging from 55 to 176 nm, from 69 to 215 nm and from 44 to 291 nm, respectively. The data from Fourier transform infrared (FTIR) spectroscopy and thermogravimetric analysis (TGA) confirmed the immobilization of essential oils into CA fibers. Moreover, the gas chromatography experiments verified the continuous release of bioactive components from CA and RO fibrous mats. Water contact angle analysis showed that electrospun fiber mats obtained by NanospiderTM technology were more hydrophilic due to the higher porosity of the mats. The electrospun CA nanofibers containing RO, CL or TH exhibited excellent antioxidant activity as shown by 2-2-diphenyl-1-picrylhydrazyl (DPPH) free radical inhibition during the period of 14 days. Furthermore, the fibrous mats exhibited antimicrobial activity, and their minimum inhibitory concentration (MIC) against *Escherichia coli*, *Pseudomonas aeruginosa* and *Listeria monocytogenes* was around 5–10 mg/mL and minimum bactericidal concentration (MBC) was around 10–15 mg/mL. In addition, the CA and CL fibrous mats proved effective in preserving the quality of fresh beef, suggesting their potential for active food packaging applications. Further studies should evaluate the practical aspects of incorporating active fibrous mats into packaging and packaging materials, including their mechanical properties, permeability characteristics, migration of bioactive compounds, long-term stability, and comprehensive interactions with food matrices.

## Figures and Tables

**Table 1:** Characteristics of electrospun CA/EO fibrous mats.

Sample	Characteristics of Electrospun Fibers				
	Concentration of EO in Fibers (wt.%)		Diameter Distribution (nm)	Average Diameter (nm)	Water Contact Angle (°)
	Theoretical	Experimental			
CA1	-	-	65–821	255 ± 25 ^A^	100 ± 2
CA1/RO10	10.80	2.01	143–992	365 ± 33 ^D^	109 ± 2
CA1/RO20	19.51	4.95	122–1754	501 ± 63 ^E^	118 ± 2
CA1/RO30	26.27	6.01	165–1561	617 ± 61 ^C^	119 ± 2
CA1/RO40	38.66	14.50	160–1849	623 ± 68 ^C^	116 ± 2
CA2	-	-	532–3896	1241 ± 114 ^A^	105 ± 2
CA2/RO10	11.29	7.44	578–2510	1242 ± 61 ^A^	110 ± 2
CA2/RO20	20.29	10.92	812–3539	1701 ± 123 ^B^	113 ± 2
CA2/RO30	26.99	11.96	685–4704	2236 ± 162 ^C^	115 ± 2
CA2/RO40	38.99	12.69	1192–4871	2939 ± 151 ^D^	108 ± 2
CA3	-	-	71–323	182 ± 11 ^D^	97 ± 2
CA3/RO40	38.89	13.00	55–176	109 ± 5 ^A^	63 ± 2
CA3/CL40	38.89	19.00	69–215	129 ± 7 ^B^	102 ± 2
CA3/TH40	38.89	14.00	44–291	147 ± 11 ^C^	45 ± 2

^A–E^: the different uppercase letters within the same column for different samples in the series show that the results are significantly different (*p* < 0.05; Duncan test).

**Table 2:** Pseudo-second-order kinetic model parameters established for the release of essential oil volatile components and residual solvents during fiber storage period of 14 days.

Volatile Component or Residual Solvent	Pseudo-Second-Order Kinetic Model		
	*q_e_* (%)	*k_2_* (%^−1^·day^−1^)	*R^2^*
Acetone	91.61	0.0621	0.9999
DCM	99.88	0.0618	0.9999
DMF	96.62	0.3444	0.9999
alpha-Pinene	53.65	0.0260	0.9759
Camphene	57.21	0.0156	0.9816
beta-Pinene	54.48	0.0263	0.9786
Eucalyptol	57.55	0.0413	0.9911
Camphor	53.32	0.0410	0.9997

**Table 3:** MIC and MBC values of different samples against *E. coli*, *P. aeruginosa* and *L. monocytogenes*.

Sample	MIC/MBC ^1^		
	*E. coli*	*P. aeruginosa*	*L. monocytogenes*
RO	112.5/250	112.5/250	112.5/375
CL	200/300	188/300	200/550
TH	100/200	100/250	250/300
CA3/RO40 fibrous mat	5/15	5/15	5/15
CA3/CL40 fibrous mat	5/10	10/15	5/10
CA3/TH40 fibrous mat	5/10	5/10	5/10

^1^ MIC and MBC values presented in μg/mL for essential oils and in mg/mL for electrospun fibrous mats.

**Table 4:** The loss *of E. coli, P. aeruginosa* and *L. monocytogenes* viability after contact with fibrous mats stored for 0, 7 and 14 days.

Sample	Storage Time (Days)	Loss of Viability (%)		
		*E. coli*	*P. aeruginosa*	*L. monocytogenes*
CA3/RO40 fibrous mat	0	15.1 ± 2.5	17.1 ± 4.7	10.5 ± 3.1
CA3/CL40 fibrous mat	0	13.2 ± 3.4	15.0 ± 4.5	8.5 ± 2.8
CA3/TH40 fibrous mat	0	98.6 ± 2.1	22.8 ± 1.8	97.1 ± 2.6
	7	99.2 ± 0.5	20.5 ± 2.6	98.3 ± 0.5
	14	96.4 ± 3.3	20.0 ± 3.0	32.1 ± 5.3

**Table 5:** Effect of the packaging on color changes of fresh beef packed under normal atmosphere during the storage at 4 ± 1 °C.

Color Parameter	Packaging	Storage Time (Days)		
		0	7	14
L*	Control	33.10 ± 1.29 ^c^	29.62 ± 1.9 ^aA^	31.01 ± 2.55 ^abcB^
	CA3/CL40	33.10 ± 1.29 ^b^	30.18 ± 3.24 ^aB^	29.10 ± 1.63 ^aA^
a*	Control	18.52 ± 0.67 ^c^	7.82 ± 0.77 ^bA^	6.40 ± 0.95 ^aA^
	CA3/CL40	18.52 ± 0.67 ^c^	12.17 ± 0.85 ^bB^	10.04 ± 0.80 ^aB^
b*	Control	14.37 ± 0.90 ^b^	8.60 ± 1.09 ^aA^	9.58 ± 1.20 ^aA^
	CA3/CL40	14.37 ± 0.90 ^b^	9.35 ± 0.64 ^aB^	10.00 ± 0.79 ^aB^
h_ab_	Control	37.78 ± 1.29 ^a^	47.62 ± 1.36 ^bB^	56.25 ± 3.78 ^cB^
	CA3/CL40	37.78 ± 1.29 ^a^	37.57 ± 2.96 ^aA^	44.90 ± 2.15 ^bA^
C*	Control	23.44 ± 0.99 ^b^	11.63 ± 1.31 ^aA^	11.55 ± 1.33 ^aA^
	CA3/CL40	23.44 ± 0.99 ^c^	15.37 ± 0.71 ^bB^	14.18 ± 0.98 ^aB^

^a–c^: the different lowercase letters within the same row for each sample show that the results are significantly different (*p* < 0.05; Duncan test); ^A,B^: the different uppercase letters within the same column for different samples show that the results are significantly different (*p* < 0.05; Duncan test).

**Table 6:** Composition and characteristics of electrospinning solutions.

Sample	Solution Properties				Characteristics	
	Concentration of CA and EO in Solution (g/L)		Solvents	Ratio of Mixed Solvents (*v*/*v*)	Conductivity (μS cm^−1^)	Shear Viscosity (mPa·s)
	CA	EO				
CA1	11	-	Acetone/DCM/DMF	2/1/1	5.76	140
CA1/RO10	11	1.33	Acetone/DCM/DMF	2/1/1	5.06	140
CA1/RO20	11	2.67	Acetone/DCM/DMF	2/1/1	4.99	130
CA1/RO30	11	4.00	Acetone/DCM/DMF	2/1/1	4.92	130
CA1/RO40	11	6.93	Acetone/DCM/DMF	2/1/1	4.92	120
CA2	11	-	Acetone/DCM	1/1	1.07	170
CA2/RO10	11	1.40	Acetone/DCM	1/1	1.05	170
CA2/RO20	11	2.80	Acetone/DCM	1/1	1.02	170
CA2/RO30	11	4.07	Acetone/DCM	1/1	0.95	170
CA2/RO40	11	7.00	Acetone/DCM	1/1	0.84	160
CA3	11	-	Acetone/DCM/DMF	2/1/1	5.76	140
CA3/RO40	11	7.00	Acetone/DCM/DMF	2/1/1	4.92	120
CA3/CL40	11	7.00	Acetone/DCM/DMF	2/1/1	7.2	140
CA3/TH40	11	7.00	Acetone/DCM/DMF	2/1/1	6.8	130

## Data Availability

Data will be made available upon request.

## Supplementary Materials

The following supporting information can be downloaded at: https://www.mdpi.com/article/10.3390/molecules31142533/s1, Figure S1: The characteristic chromatograms of clove essential oil (top), rosemary essential oil (middle), and thyme essential oil (bottom). Peaks marked by numbers in top chromatogram: 1—eugenol, 2—caryophyllene, 3—eugenyl acetate; in middle chromatogram: 1—alpha-pinene, 2—camphene, 3—beta-pinene, 4—beta-myrcene, 5—p-cymene, 6—limonene, 7—eucalyptol, 8—camphor, 9—isoborneol, 10—borneol, 11—terpineol, 12—bornyl acetate; in bottom chromatogram: 1—alpha-pinene, 2 —camphene, 3—beta-myrcene, 4—p-cymene, 5—eucalyptol, 6—gamma-terpinene, 7—linalool, 8—camphor, 9—terpinen-4-ol, 10—thymol, 11—carvacrol.

## Abbreviations

The following abbreviations are used in this manuscript:

CA	Cellulose acetate
RO	Rosemary essential oil
CL	Clove essential oil
TH	Thyme essential oil
PP	Polypropylene
Ph Eur	European Pharmacopoeia
FTIR	Fourier transform infrared
TG	Thermogravimetric
DPPH	2-2-Diphenyl-1-picrylhydrazyl
DCM	dichloromethane
DMF	N,N-dimethylformamide
MIC	Minimum inhibitory concentration
MBC	Minimum bactericidal concentration
EO	Essential oil
SEM	Scanning electron microscope
TIC	Total ion chromatographic
GC-MS	Gas chromatography–mass spectrometry

## References

[1] Xue J., Wu T., Dai Y., Xia Y. (2019). Electrospinning and electrospun nanofibers: Methods, materials, and applications. Chem. Rev..

[2] Acuna D., Cohn N., Quero F. (2023). Electrospun bioactive tertiary glass nanoparticles-containing silica/gelatin/polyethylene oxide hybrid membranes for potential dental bone tissue engineering applications. Mater. Lett..

[3] Wei H., Wen J., Yan S., Zhang H., Liu Y., Xia Y., Li J., Cao R., Zhu M. (2025). Adjusting morphologies of wound dressing by transferring skin textures through electrospinning technology. Colloids Interface Sci. Commun..

[4] Ao F., Yin C., Luo X., Shen W., Ge X., Zheng Y. (2025). Controlled dual drug delivery system based on gelatin electrospinning membranes for wound healing promotion. Int. J. Biol. Macromol..

[5] Jankowska K., Su Z., Jesionowski T., Zdarta J., Pinelo M. (2023). The impact of electrospinning conditions on the properties of enzymes immobilized on electrospun materials: Exploring applications and future perspectives. Environ. Technol. Innov..

[6] Zhang Y., Min T., Zhao Y., Cheng C., Yin H., Yue J. (2024). The developments and trends of electrospinning active food packaging: A review and bibliometrics analysis. Food Control.

[7] Dey A., Neogi S. (2019). Oxygen scavengers for food packaging applications: A review. Trends Food Sci. Technol..

[8] Khaneghah A.M., Hashemi S.M.B., Limbo S. (2018). Antimicrobial agents and packaging systems in antimicrobial active food packaging: An overview of approaches and interactions. Food Bioprod. Process..

[9] Liu Y., Li L., Yu Z., Ye C., Pan L., Song Y. (2023). Principle, development and application of time–temperature indicators for packaging. Packag. Technol. Sci..

[10] Wei H., Seidi F., Zhang T., Jin Y., Xiao H. (2021). Ethylene scavengers for the preservation of fruits and vegetables: A review. Food Chem..

[11] Lee D.S., Wang H.J., Jaisan C., An D.S. (2022). Active food packaging to control carbon dioxide. Packag. Technol. Sci..

[12] Khalf A., Madihally S.V. (2017). Recent advances in multiaxial electrospinning for drug delivery. Eur. J. Pharm. Biopharm..

[13] Wen P., Wen Y., Zong M., Linhardt R.J., Wu H. (2017). Encapsulation of bioactive compound in electrospun fibers and its potential application. J. Agric. Food Chem..

[14] Xia Q., Chen C., Yao Y., Li J., He S., Zhou Y., Li T., Pan X., Yao Y., Hu L. (2021). A strong, biodegradable and recyclable lignocellulosic bioplastic. Nat. Sustain..

[15] Li T., Chen C., Brozena A.H., Zhu J.Y., Xu L., Driemeier C., Dai J., Rojas O.J., Isogai A., Wågberg L. (2021). Developing fibrillated cellulose as a sustainable technological material. Nature.

[16] Clarkson C.M., El Awad Azrak S.M., Forti E.S., Schueneman G.T., Moon R.J., Youngblood J.P. (2021). Recent developments in cellulose nanomaterial composites. Adv. Mater..

[17] Han S.O., Youk J.H., Min K.D., Kang Y.O., Park W.H. (2008). Electrospinning of cellulose acetate nanofibers using a mixed solvent of acetic acid/water: Effects of solvent composition on the fiber diameter. Mater. Lett..

[18] Tungprapa S., Puangparn T., Weerasombut M., Jangchud I., Fakum P., Semongkhol S., Meechaisue C., Supaphol P. (2007). Electrospun cellulose acetate fibers: Effect of solvent system on morphology and fiber diameter. Cellulose.

[19] Liu H., Hsieh Y.L. (2002). Ultrafine fibrous cellulose membranes from electrospinning of cellulose acetate. J. Polym. Sci. Part B Polym. Phys..

[20] Celebioglu A., Uyar T. (2011). Electrospun porous cellulose acetate fibers from volatile solvent mixture. Mater. Lett..

[21] Ma Z., Ramakrishna S. (2008). Electrospun regenerated cellulose nanofiber affinity membrane functionalized with protein A/G for IgG purification. J. Membr. Sci..

[22] Liao N., Unnithan A.R., Joshi M.K., Tiwari A.P., Hong S.T., Park C.H., Kim C.S. (2015). Electrospun bioactive poly (ε-caprolactone)–cellulose acetate–dextran antibacterial composite mats for wound dressing applications. Colloids Surf. A Physicochem. Eng. Asp..

[23] Sethunga M., Gunathilake K.D.P.P., Ranaweera K.K.D.S., Munaweera I. (2024). Antimicrobial and antioxidative electrospun cellulose acetate-essential oils nanofibrous membranes for active food packaging to extend the shelf life of perishable fruits. Innov. Food Sci. Emerg. Technol..

[24] Ullah A., Saito Y., Ullah S., Haider M.K., Nawaz H., Duy-Nam P., Kharaghani D., Kim I.S. (2021). Bioactive Sambong oil-loaded electrospun cellulose acetate nanofibers: Preparation, characterization, and in-vitro biocompatibility. Int. J. Biol. Macromol..

[25] Wsoo M.A., Razak S.I.A., Bohari S.P.M., Shahir S., Salihu R., Kadir M.R.A., Nayan N.H.M. (2021). Vitamin D3-loaded electrospun cellulose acetate/polycaprolactone nanofibers: Characterization, in-vitro drug release and cytotoxicity studies. Int. J. Biol. Macromol..

[26] Rana R., Gill A.S., Deol P.K., Kaur I.P. (2024). Investigation of cellulose acetate electrospun films for controlled drug permeability. J. Drug Deliv. Sci. Technol..

[27] Bakkali F., Averbeck S., Averbeck D., Idaomar M. (2008). Biological effects of essential oils—A review. Food Chem. Toxicol..

[28] Sánchez-González L., Vargas M., González-Martínez C., Chiralt A., Cháfer M. (2011). Use of essential oils in bioactive edible coatings: A review. Food Eng. Rev..

[29] Ricardo-Rodrigues S., Rouxinol M.I., Agulheiro-Santos A.C., Potes M.E., Laranjo M., Elias M. (2024). The antioxidant and antibacterial potential of thyme and clove essential oils for meat preservation—An overview. Appl. Biosci..

[30] Hofmeisterová L., Bajer T., Walczak M., Šilha D. (2024). Chemical composition and antibacterial effect of clove and thyme essential oils on growth inhibition and biofilm formation of *Arcobacter* spp. and other bacteria. Antibiotics.

[31] Kraśniewska K., Gniewosz M. (2025). Active packaging based on a PET/PP food-grade film coated with pullulan and clove essential oil: Physicochemical and antimicrobial properties. Molecules.

[32] Navikaite-Snipaitiene V., Ivanauskas L., Jakstas V., Rüegg N., Rutkaite R., Wolfram E., Yildirim S. (2018). Development of antioxidant food packaging materials containing eugenol for extending display life of fresh beef. Meat Sci..

[33] Nazari M., Majdi H., Gholizadeh P., Kafil H.S., Hamishehkar H., Zarchi A.A.K., Khoddami A. (2023). An eco-friendly chitosan/cellulose acetate hybrid nanostructure containing *Ziziphora clinopodioides* essential oils for active food packaging applications. Int. J. Biol. Macromol..

[34] Teixeira R.F., Filho C.A.B., Borges C.D. (2022). Essential oils as natural antimicrobials for application in edible coatings for minimally processed apple and melon: A review on antimicrobial activity and characteristics of food models. Food Packag. Shelf Life.

[35] Liakos I.L., Holban A.M., Carzino R., Lauciello S., Grumezescu A.M. (2017). Electrospun fiber pads of cellulose acetate and essential oils with antimicrobial activity. Nanomaterials.

[36] Spasova M., Stoyanova N., Stoilova O. (2024). Electrospun materials based on cellulose acetate loaded with rosmarinic acid with antioxidant and antifungal properties. Biomimetics.

[37] Chen W., Zhao P., Yang Y., Yu D.-G. (2023). Electrospun beads-on-the-string nanoproducts: Preparation and drug delivery application. Curr. Drug Deliv..

[38] Partheniadis I., Nikolakakis I., Laidmäe I., Heinämäki J. (2020). A mini-review: Needleless electrospinning of nanofibers for pharmaceutical and biomedical applications. Processes.

[39] Vass P., Szabó E., Domokos A., Hirsch E., Galata D., Farkas B., Démuth B., Andersen S.K., Vigh T., Verreck G. (2020). Scale-up of electrospinning technology: Applications in the pharmaceutical industry. WIREs Nanomed. Nanobiotechnol..

[40] Omer S., Forgách L., Zelkó R., Sebe I. (2021). Scale-up of electrospinning: Market overview of products and devices for pharmaceutical and biomedical purposes. Pharmaceutics.

[41] Tarhan I. (2021). A robust method for simultaneous quantification of eugenol, eugenyl acetate, and β-caryophyllene in clove essential oil by vibrational spectroscopy. Phytochemistry.

[42] Navikaite-Snipaitiene V., Rutkaite R., Ivanauskas L., Jakstas V., Fieseler L., Rüegg N., Yildirim S. (2025). Development of thyme essential oil-based coatings and assessment of their antimicrobial activity. Appl. Food Res..

[43] Wang H.F., Yih K.H., Huang K.F. (2010). Comparative study of the antioxidant activity of forty-five commonly used essential oils and their potential active components. J. Food Drug Anal..

[44] Faustman C., Sun Q., Mancini R., Suman S.P. (2010). Myoglobin and lipid oxidation interactions: Mechanistic bases and control. Meat Sci..

[45] Matulevicius J., Kliucininkas L., Martuzevicius D. (2014). Electrospinning of cellulose acetate fibers from a ternary solvent system. Chemija.

[46] Alaraj M., Ren Z.J., Park J.D. (2014). Microbial fuel cell energy harvesting using synchronous flyback converter. J. Power Sources.

[47] Arshak K.I., Almukhtar B. (2000). The design and development of a novel flyback planar transformer for high frequency switch mode DC–DC converter applications. Microelectron. J..

[48] Sutka A., Kukle S., Gravitis J., Milašius R., Malašauskienė J. (2013). Nanofibre electrospinning poly(vinyl alcohol) and cellulose composite mats obtained by use of a cylindrical electrode. Adv. Mater. Sci. Eng..

[49] Barbosa R.F.S., Yudice E.D.C., Mitra S.K., Rosa D.S. (2021). Characterization of rosewood and cinnamon cassia essential oil polymeric capsules: Stability, loading efficiency, release rate and antimicrobial properties. Food Control.

[50] Wen P., Zhu D.H., Wu H., Zong M.H., Jing Y.R., Han S.Y. (2016). Encapsulation of cinnamon essential oil in electrospun nanofibrous film for active food packaging. Food Control.

[51] Rieger K.A., Schiffman J.D. (2014). Electrospinning an essential oil: Cinnamaldehyde enhances the antimicrobial efficacy of chitosan/poly(ethylene oxide) nanofibers. Carbohydr. Polym..

