# Supplementary material for: Preparation and Properties of Electrospun Cellulose Acetate Fibers Containing Rosemary, Clove, and Thyme Essential Oils
Source: Molecules. 2026 Jul 21;31(14):2533. doi: 10.3390/molecules31142533 (PMC13415212; doi:10.3390/molecules31142533)
Supplement: Supplementary file 1 [file molecules-31-02533-s001.zip › molecules-4380707-supplementary.pdf]

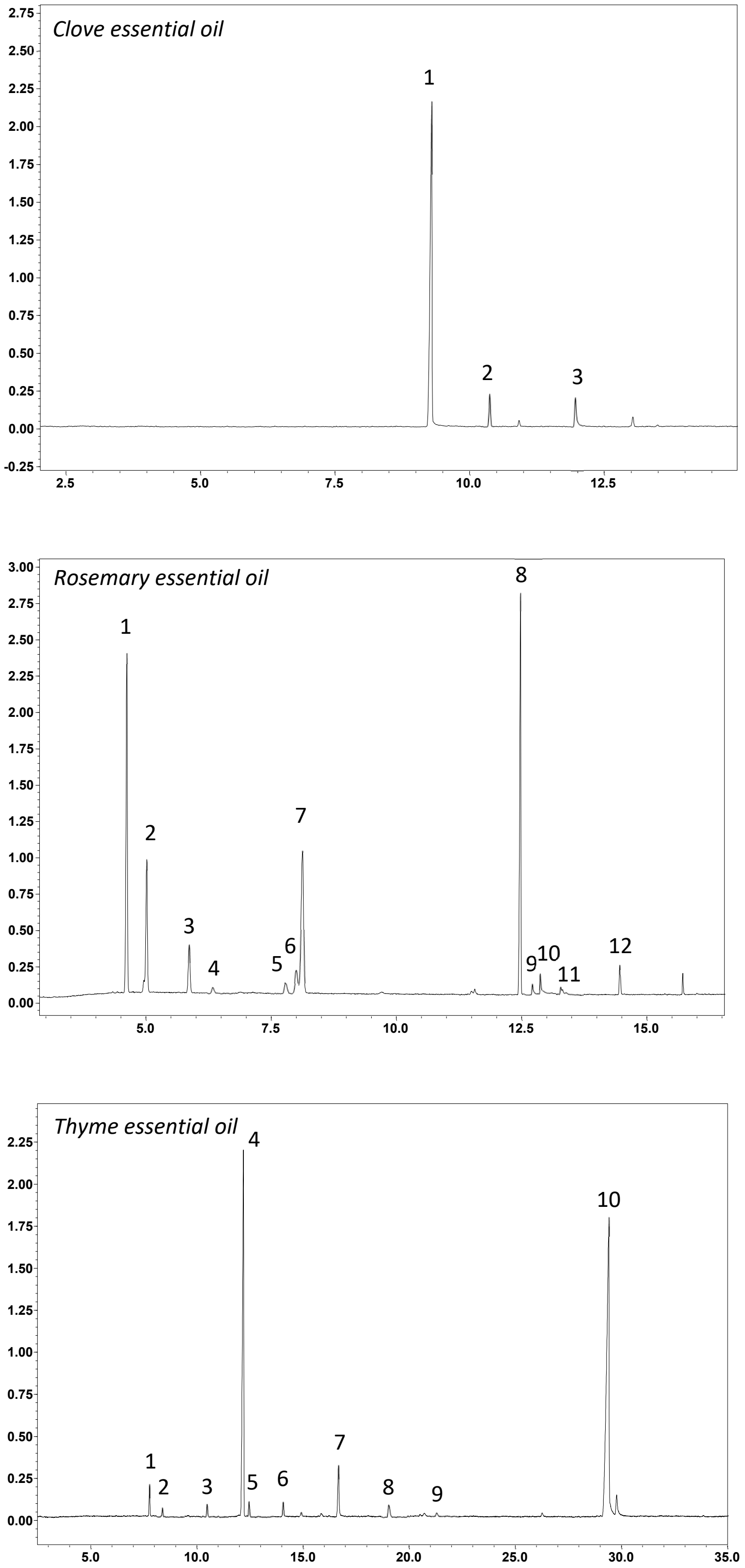

Figure S1. The characteristic chromatograms of clove essential oil (top), rosemary essential oil (middle), and thyme essential oil (bottom). Peaks marked by numbers in top chromatogram: 1 - eugenol, 2 - caryophyllene, 3 - eugenyl acetate; in middle chromatogram: 1 - alpha-pinene, 2 - camphene, 3 - beta-pinene, 4 - beta-myrcene, 5 - p-cymene, 6 - limonene, 7 - eucalyptol, 8 - camphor, 9 - isoborneol, 10 - borneol, 11 - terpineol, 12 - bornyl acetate; in bottom chromatogram: 1 - alpha-pinene, 2 - camphene, 3 - beta-myrcene, 4 - p-cymene, 5 - eucalyptol, 6 - gamma-terpinene, 7 - linalool, 8 – camphor, 9 - terpinen-4-ol, 10 - thymol, 11 - carvacrol.
